# Implementation of preventive strength training in residential geriatric care: a multi-centre study protocol with one year of interventions on multiple levels

**DOI:** 10.1186/1471-2318-9-51

**Published:** 2009-11-24

**Authors:** Michael Brach, Frank Nieder, Ulrike Nieder, Heinz Mechling

**Affiliations:** 1 Institute of Sport Science, University of Münster, Horstmarer Landweg 62b, 48149 Münster, Germany; 2 Institute of Sport Gerontology, German Sport University Cologne, Am Sportpark Müngersdorf 6, 50933 Cologne, Germany

## Abstract

**Background:**

There is scientific evidence that preventive physical exercise is effective even in high age. In contrast, there are few opportunities of preventive exercise for highly aged people endangered by or actually in need of care. For example, they would not be able to easily go to training facilities; standard exercises may be too intensive and therefore be harmful to them; orientation disorders like dementia would exacerbate individuals and groups in following instructions and keeping exercises going. In order to develop appropriate interventions, these and other issues were assigned to different levels: the individual-social level (ISL), the organisational-institutional level (OIL) and the political-cultural level (PCL). Consequently, this conceptional framework was utilised for development, implementation and evaluation of a new strength and balance exercise programme for old people endangered by or actually in need of daily care. The present paper contains the development of this programme labeled "fit for 100", and a study protocol of an interventional single-arm multi-centre trial.

**Methods:**

The intervention consisted of (a) two group training sessions every week over one year, mainly resistance exercises, accompanied by sensorimotor and communicative group exercises and games (ISL), (b) a sustainable implementation concept, starting new groups by instructors belonging to the project, followed by training and supervision of local staff, who stepwise take over the group (OIL), (c) informing and convincing activities in professional, administrative and governmental contexts, public relation activities, and establishing an advisory council with renowned experts and public figures (PCL). Participating institutions of geriatric care were selected through several steps of quality criteria assessment. Primary outcome measures were continuous documentation of individual participation (ISL), number of groups continued without external financial support (at the end of the project, and after one year) (OIL). Secondary outcome was measured by sensorimotor tests and care-related assessments in the beginning and every 16 weeks (ISL), by qualitative outcome descriptions 12 months after group implementation (OIL) and by analysis of media response and structured interviews with stakeholders, also after 12 months (PCL).

**Conclusion:**

Exemplarily, preventive exercise has been established for a neglected target population. The multi-level approach used here seems to be helpful to overcome institutional and individual (attitude) barriers.

**Trial registration:**

Current Controlled Trials ISRCTN55213782

## Background

Successful ageing is both in a psychological and physiological dimension a main goal most elderly people aim for. This includes keeping one's independence [1,2]. Generally, it is well-known that aerobic and strength training can effectively contribute to this aim, even in high age [3]. However, highly aged people may have problems to easily go to training facilities; standard exercises may be too intensive and therefore be harmful to them, or too complex for effective and save execution; standard test procedures may not be appropriate for this target group; trainers may be inadequately educated. Additionally, the increased prevalence and incidence of orientation disorders like dementia would exacerbate individuals and groups in following instructions and keeping exercises going on. These general preconditions are even more precarious for people in need of ambulant or residential care. Therefore, research for this target group should not focus on strength and balance training alone, but also on how to implement programmes and how to distribute good practice to the public. In this paper, the following sections contain a rough problem analysis leading to multi-level approaches of programme development, implementation and diffusion. A preventive training programme and a study protocol will be described. An outlook of planned evaluation is given.

### Problem analysis and conceptual framework

Reconsidering the introductorily mentioned preconditions, some aspects to be met are at the individual level, like selecting proper exercises or handling deficits in short time memory, or in spacial and temporal orientation problems within the target group. Benefits like bindingness, motivation and training quality, gained by dated exercises in a group, led by an instructor, are at the social level. Especially the social contacts meet loneliness, which is often observed in residential care facilities, even though community activities exist. On the other hand, usually in a group there are individual differences in performance, understanding, and needs. An educated and experienced group instructor should be used to managing these issues. The examples show that the social level interferes with the individual level. Therefore in this paper the domain will be referred to as *individual-social level *(ISL).

Other preconditions mentioned in the introduction are due to the institution the residents live in. For example, the reader may consider an 82-year-old woman living in residential care. She may be known for needing some help in personal mobility, for whatever reason. A nurse or an assistant is in charge to provide the help needed, for example to guide her to her room, or to push the wheel chair, or just to remember and to invite her to the training session. If there were any recurrent problems for her reaching the training room in time, these would not be located at the individual level, but at the *organisational or institutional level *(OIL): the assistant may be busy with another task, the care unit may be understaffed or the staff may take the training programme not as serious as they should.

Trying to implement training groups soon leads to another level of issues. Living in residential care, for example, would leave most potential participants only a small pocket money. Therefore, obligation to pay the full costs of training themselves raises a high barrier to most residents. On the other hand, directors of care homes complained being compelled to bring down care staff during the past years, due to the economic situation in geriatric care in Germany [4,5]. Therefore, even being convinced of the usefulness of preventive training does not mean that there would be enough financial resources to implement it. At the care and health system level, geriatric care is often thought to be placed at the end of some continuum with prevention at its opposite end. This seems to reflect the opinion of many professionals in the field as well as parts of the public. In this paper, we assign the system-related issues to the *political and cultural level *(PCL). In summary, different levels of issues and interventions are playing roles when preventive training in residential care is to be developed and implemented:

• the individual-social level (ISL)

• the organisational-institutional level (OIL) and

• the political-cultural level (PCL).

Using this framework may help to meet an approach labeled *theory of the problem *and *theory of the intervention *[6,7], for both parts relate to several levels. Consequently, these levels should also be considered in the evaluation of the programme. Multi-level evaluations were carried out in a number of other social programmes [8,9], and were recommended for physical exercise [[10], pp. 214-220]. In the subsection Recruitment, inclusion and exclusion at the institutional and at the individual level (Methods), the corresponding procedure of the present project will be described.

### Aims, approaches and strategies of the over-all programme

Starting in 2004, a multiprofessional team from sport and social sciences, scholars and practitioners, ventured on a mission according to the motto *fit for 100*. The ongoing tasks are:

• to conceptualize a scholarly-based strength and balance training programme targeting on very old persons endangered by, or actually in, need of care.

• to conduct this programme for the purpose of research and for developing good practice models.

• to initiate broad diffusion and establishment of the programme in institutions providing geriatric care.

The activities were based on two prerequisites on the role of science and academia: Firstly, the project was mainly to apply scientific knowledge to a practice field. As usual in engineering, on the course questions would arise to be answered using scientific methods [11]. Secondly, scientists and the university institute would be responsible for initiation, development and quality management, but not for distribution and maintenance. On the long run, a specific organisation taking over these tasks has to be joined or to be founded.

In consequence, all activities were planned at a multi-level scale according to the previous section. From the beginning, aspects of structural development and sustainment were considered. Therefore, the development and dissemination strategy was determined to start with residential geriatric care. The aims of this strategy were to show that

• the need of assistance with daily activities is no barrier to do physical exercise successfully (ISL).

• within the complex environment of a residential care institution the temporal, spatial, personal and financial means to set up a high-quality training programme and to continuously conduct it can be provided (OIL).

• the constructs prevention and geriatric care do not exclude each other (PCL).

For the overall *fit for 100 *mission, several stages were planned:

• General conceptualisation and fundraising (06-2004 until 05-2005): A strategic cooperation with the State Seniors' Agency of North-Rhine Westphalia (German: *Landesseniorenvertretung*) and with the State Sport Federation of North-Rhine Westphalia (*Landessportbund*) was established. A successful application for a grant on a social affairs model project (*sozialpolitisches Modellprojekt*) in the area of geriatric care allowed to enter stage I.

• Stage I - Sustainable local models (06-2005 until 07-2008): The exercise programme is developed, feasibility and adoption are tested. The details are described in the present paper.

• Stages II and III - Dissemination and research (12-2006 until 03-2009). The preparation for a state-wide dissemination of the programme includes financial (grant applications), organisational (test of the dissemination concept) and personnel aspects. In addition, an extension of the programme for demented people is tested within a time series approach. Conception and results of these stages will be published elsewhere.

In the last two parts of the background section, the focus and principles of the exercise programme are laid down. First, a literature overview is given, covering exercise for frail and highly-aged people. Concrete reference programmes are discussed. On this base the new exercise programme is developed. Its content and organisation are described in the last subsection, including important conditions for participants and instructors.

### Literature on exercise with frail and highly-aged subjects

The following three subsections present a concise literature overview on effects, configuration and organisation of training with very aged people. It will help to choose and to justify goals and exercises. In order to learn from experiences with the implementation of programmes in the targeted setting, the forth subsection focuses on German circumstances.

#### Training effects in high age

Seven exercise programmes, called the FICSIT studies (*Frailty and Injuries: Cooperative Studies of Intervention Techniques*), were carried out at seven different places in the United States. They focused on endurance, balance and flexibility training, Tai Chi, resistance training and training for functional activities, multifactorial und individualized training including medication changes and behaviour aspects, behaviour accommodation, and educational intervention, respectively. Province et al. [12] did a meta-analysis on these programmes and concluded that non-directed and non-specific exercises had no effects on well-being of the aged. All interventions utilising physical activity were successful.

It is important to know, however, which kind of physical activity is useful. Via strength training with dumb-bells and cuffs with weights, aged people between 73 and 94 years achieved significant improvements regarding their efficiency in balance, stair climbing, 6-meter-gait and chair-rising test [13]. For the over-75-years-old, Gill et al. [14] describe the effectiveness of daily balance-training and flexibility exercises in combination with training for the upper and lower part of the body. Three times a week, exercises were performed using an elastic band in a sitting position. In the training group, the onset of ADL reduction was seen later than in the control group, and the amount was lower. Hruda et al. [15] report increased muscle strength and improved performance of functional activities through progressive training for the lower part of the body. The subjects were 75 to 94 years old. This training programme consisted of exercises for strength and functional performance.

The positive effects of training in endurance and strength for the frail and the very old have been known for decades: for instance, Liesen et al. [16] demonstrated a gain of maximum oxygen uptake () in 55 to 70-year-old untrained people by eight weeks of endurance training. Delorme [17] displayed the benefits of training with high resistance and the importance of such a programme especially for recreation of functionality after injury. Significant gains in muscle strength, muscle size and functional mobility through highly intensive resistance training were detected by Fiatorone et al. [18] even for people over 90 years living in nursing homes. Sullivan et al. [19] support progressive resistance training actually for frail elderly who are still recovering from disease.

In conclusion, resistance training can yield positive effects even for frail and highly-aged people. Type and magnitude of such effects depend on certain parameters, e.g. choice and order of exercise, intensity, number and length of sets, time under tension and rest length period [20,21]. Does intensity increase over time? Which movement instruction is given? Is there any feedback or correction for the participants? These and more aspects also determine training outcomes. Unfortunately, many papers on interventional trials only partially contain this important information [14,22-25].

#### Training configuration: frequency and intensity

Exercise intensity and number of exercises per week vary between programmes. Training frequency varies between two and seven workouts per week. Mostly three days of resistance training per week had to be performed [15,19,25-31]. Exercise intensity spanned from low [22], moderate [32] up to progressive increasing [15,19] or high intensity [18,24,33].

In sport science, strength training intensity is often described in relation to the one repitition maximum (1RM). 1RM is defined as the maximum load under which a subject is able to correctly perform a specific exercise for one time, but not for a second time. Seynnes and colleagues [31] compared several trials and found that training with high intensity (80% of 1RM) is as save as training with low intensity (40% of 1RM), while - as expected - high-intensity training is considerably more effective concerning physiological and functional measures. Effects of the eccentric phase with resistance training for the lower extremity were considered in an investigation of LaStayo et al. [29]. The participants of the eccentric (negative work) resistance exercise showed larger force increases than the participants of the traditional weight training resistance exercise with free weights and weight machines. Therefore, the eccentric phase in the new programme is enlarged to four seconds (see below).

#### Training organisation: social forms and guidance

Group-based and home-based training programmes differ in the number of weekly training units and in the possibilities to observe participance and performance. The participants of Campbell's study [23] had to train six times a week, at Gill et al. [14] even daily. McCool & Schneider [30] conducted only three exercise units per week, but with three high-intensity sets and with guidance of a physiotherapist at the beginning. Timonen et al. [34] showed that group exercises had a significant advantage in mood improvement compared with exercising at home; Binder et al. [22] reported significantly larger improvements in three of four output measurements with an exercise training programme opposite to a home exercise programme. Supervised training is more effective than independent training at home. Brill et al. [13] described significant improvements of "functional performance" (timed chair stand, stair climb, 6-meter walk, balance) with a strength training using ankle weights and dumb bells led by an instructor, at Boshuizen et al. [26] the more guided group (two supervised group sessions and one unsupervised home session) had a tendency to better results than the group with less guidance (one supervised group session and two unsupervised home sessions) and the control group. Extensive studies are needed to clarify whether these tendencies subsist.

Other arguments seem to be more important for decisions on training organisation: while Hinrichs and colleagues [35], for example, focus on home-based training in order to reach their target group more easily, the present study prefers group sessions for social interaction and instructions.

#### Training implementation: example programmes from targeted settings

Becker et al. [36] conducted a programme which was later referred to as *Ulm model*. Within a group offer for six to eight persons in residential geriatric care settings, strength and balance exercises were carried out at least once per week. In addition, hip protectors, advice for the residents, their relatives and for the caregivers as well as a modification of environment were offered. The exercise programme included ten exercises (75% of 1 RM) for around 75 minutes. Arm and trunk exercises in a seated position alternated with leg exercises in a standing position. Due to this alternation of standing and sitting positions, an additional training stimulus was obtained.

A multifactorial evaluation (cluster randomized, *n *= 981) was carried out between 1998 and 2000. In the experimental group, the number of falls was 40% lower compared to the control group. The number of persons who experienced multiple falls was 44% lower [37]. The exercise programme of the Ulm model was disseminated state-wide and adopted by other German states, in the fields of residential care and ambulatory care [38,39].

Similiar to the Ulm model, an exercise programme called PATRAS (*Paderborn training study for seniors *[40,41]) was conducted twice per week during a period of 16 weeks with 46 residents of three nursing care institutions in Paderborn, Germany. In comparison to the Ulm model, PATRAS contained three additional exercises (grip strength, lower arm exercise and seated knee extension), but there were no squatting exercises. In addition, there was another sequence of exercise: at first, all seated exercises for arms and torso, and the knee extension exercise were executed. Subsequently, all leg exercises were performed in a standing position. Instead of dumb bells, as used in Ulm, small bags filled with weight balls were used for the arm exercises.

Tittlbach et al. [42] accomplished a psychomotor training programme with residents of nursing homes. They showed significant positive effects on coordination, flexibility and cognitive functions but not on ADL and strength. Schumann-Schmid and Dittmar [43] reported on another intervention scheme with strength, flexibility, balance, body perception and memory exercises. These were offered in five nursing and care homes in the city of Mainz, Germany. The training duration and frequency was 30 minutes, three to five times a week. At present, results are not yet available.

Official publications of these programmes as well as personal communications with the authors and colleagues exerted influence on the development of the new programme, as shown in the following sections.

### Development of the new exercise programme

#### Overview and principles

Based on the reviews given above, the new programme has been developed. While the reference programmes aimed, for example, at reduction of fall risks and improving nutritional status, the new programme concentrates on maintaining or re-establishing everyday life competence and mobility. Therefore, the following principles have been set up:

• Orientation towards programme goals: training of muscle groups involved in activities of daily living. Enhancing strength, balance, and flexibility, in order to support independent daily activities, fall prevention, and well-being.

• Orientation towards principles of modern training science: regular training, twice per week for 60 minutes, exercises with an intensity of approximately 80% of 1RM, ten repetitions performed emphasing the eccentric contraction. Professional guidance, increasing load, individualisation. Motivation for self-sustaining participation by group exercise.

• Accordance with the abilities and needs of the target group: every participant should experience his or her own motivating success by reaching individual goals. Group exercise as social event. Fewest possible exclusion criteria.

In summary, a goal-oriented, science-oriented and target-group-oriented exercise programme has been created. Table 1 summarises the main training characteristics. Referring to the conceptual framework introduced above, this exercise programme relates to the individual-social level. The instructor would act at the same level, but is also an interface to the institutional level.

**Table 1:** Characteristics of the new exercise programme. Modified according to Mechling und Brach [[48], p. 15].

Focus	Strength, balance, ability to perform ADL
Exercise choice	10 strength exercises supporting everyday competence Sensorimotor exercise and games during opening and end phase of the session
Exercise frequency	Group sessions twice a week
Exercise volume	1-2 sets with 10 repetitions each
Duration of one session	45 to 60 minutes
Exercise intensity	80% of 1RM, controlled by Borg's rating scale of perceived exertion
Minimum requirements	Ability to stand or ability to perform the alternative exercises Ability to join group activities
Instructors	Instructors undergo particular programme training
Participation	Regular and continuous exercise is essential in order to gain desired effects
Additional care	Additional assistant is present to care for frail and for disorientated participants
Liquid intake	Breaks with beverage offers are an integrant part of the training intervention

#### Exercises

The main part of the new training programme contains ten resistance exercises, covering muscle groups neccessary for everyday movements according to the programme focus (see table 2). One or two series with a set of ten repetitions of each exercise are carried out in a slow, controlled manner with one minute active break after each set and between the series. During the opening and closing phases, sensorimotor and communicative group exercises and games are conducted to enhance motivation and social dynamics. As discussed in the literature overview, exercises for upper and lower body or extremities are alternating. This way, standing up and sitting down is utilised as part of the training and the participants will get used to it (again) as an everyday movement.

**Table 2:** Muscle groups, daily activities and corresponding exercises. Translated and extended from the *fit for 100 *manual [[66], p. 8].

**Muscle group**	**Motor activity**	**Corresponding example of daily activity**	**Corresponding example exercise from the exercise programme**
			
Spinal erector	Trunk erection	Upright posture	To consciously put upright the trunk
Upper back muscles	Trunk erection	Deep respiration	Extend arms toward the ceiling (exercise *winner*)
Hamstring and glutei muscles	Hip extension	Rising from a chair, walking safely, postural stability	Light squats
Quadriceps and hip flexor muscles	Leg extension and hip flexion, respectively	Rising up, walking, climbing stairs	Extension phase squats (exercise *treading water*)
Foot flexion, foot extension, calves	Foot and ankle stabilisation during walking	Walking safely, overcoming trip hazards	Sitting or standing position: exercise *heel and tiptoeing*
Muscles of the shoulder	Arm rising	All movements using upper extremities, e.g. combing, hairdressing, personal hygiene, dressing, eating, drinking	Arm lifting towards ceiling, exercises *bird *and *winner*
Chest, shoulder and arm muscles	Support movements with arms	Lifting and carrying objects, hanging up laundry, putting on elasticized stockings	Exercises *butterfly, nutcracker *and *biceps*
Lower arm and hand muscles	Movements of hand and fingers	Squeezing a flannel, opening a bottle, moulding coffee, cutting bread, eating	Coordinative finger exercises using weight pouches; optional weight exercises *motorcycle driving *and *paste *rolls
Abductors and small glutei muscles, hip joint muscles	Sideward lift of the extended leg (alternatively)	Lateral balance while standing and walking	Lateral hip elevators
Abdominal, trunk and pelvic floor muscles	Keep trunk tension	Upright posture	Sitting position: squeeze ball or cushion between the legs; pressure with both legs against the ground

Training intensity should be about 80% 1RM. This can be achieved by the use of ankle weights containing an individual and changeable number of weight bars, and the use of different dumb-bell weights. Intensity is controlled by perceived exertion (see next subsection for details).

#### Target group orientation and staff

Inclusion prerequisites for participants have been set as low as possible: she or he has to be able to act in a group and stand on her/his feet for a while. Each group is allowed to include few demented people or wheel chair users. Local staff is instructed to invite residents to the exercises, focussing on persons with problems in mobility. Participation is voluntary and can be stopped every time. New volunteers are invited to take the place of drop-outs.

In every way, the needs and abilities of the heterogeneous group of old and very old persons should be met. Therefore, an additional assistant on the institution's staff was present in each group. Need for special assistance could arise from a dropped training device, from hearing or understanding problems, or from a sudden need for guidance to the lavatory. In most cases of assistant activities, the instructor would go on and nobody would mind - in avoidance of embarrassment. For wheelchair users, alternative exercises have been prepared. Cognitive limited persons would be able to train in segregative as well as integrative groups with more intensive training support.

The instructor would observe and assist the exercise and would provide visual, verbal and tactile correction and motivation. She or he should stimulate an intensity between "somewhat hard" and "hard (heavy)" on Borg's perceived exertion scale [44], corresponding to a point of instantaneous muscular fatigue at the end of a certain exercise. As Löllgen [45] describes, Borg's scale gives comprehensible and coherent references for exercise intensity in primary and secondary prevention, also for resistance training. While generally this scale is easy to use, it may confuse very old participants. Therefore, the criterion for reaching an appropriate load in strength exercise was stated "feels bold strain within the muscles, which would not be present during everyday movements".

The participants are expected to behave self-consciously in a new group, and to have problems to execute new movements immediately. Therefore, during the first weeks, learning the exercises and familiarising with the group are the main tasks of the session. At an early stage, the duration of a session should be limited to 30 or 45 minutes. At later stages, the training should be expanded to 60 minutes.

The description above shows that the instructor is a key to the success of each local group via his or her ability to judge the personal differences of the members of the group. Knowledge and judgement regarding exercise instruction and interaction with multimorbid and frail elderly, associated with personal properties, are essential for leading groups in the present programme. Consequently, emphasis must be given to the training of the instructors by a special conception. The licencing conditions include full three day training, an on-site visitation, and advanced training as an update every two years. The conception will be described elsewhere, in context with a study on instructor behaviour, which has been conducted already.

## Methods

### Overview: Conception and design of the study

The exercise programme described in the subsection Intervention is oriented towards a broad target group of elderly, who are frail or in danger of frailty. The development and dissemination strategy intends residential geriatric care to be a practical starting point to reach parts of the target group (facilities, staff, short transport). With these aims in mind (see also subsection Aims, approaches and strategies (Background)), the success of the first steps would strongly depend on the concrete institutions where the programme would be implemented initially. Therefore - in contrast to the broad inclusion at the individual level - a multistep selection process is conducted in order to find "optimal" institutions. "Optimal" means that the choice should give reason for a high probability of the institutions

• to be able to conduct the programme for at least one year,

• to adopt the programme before the project phase (and funding) ends, and

• to serve as a model for other institutions having a similar structure, organisation, or environment, including possible problems to be solved in maintaining the programme.

The selection procedure is laid down in the following subsection, in order to find optimal model institutions. Unlike random sampling, these institutions will not represent a population of all institutions being part of the audience for the programme. In consequence, it is not indicated to draw statistical conclusions from the model institutions to a certain population. Inferential statistics will be part of later phases of the project, which will deal with dissemination of the programme. For the present study is oriented towards development, feasibility and best practice modeling, data analyses will be imbedded in a framework of evaluation research (see subsection Data analyses and evaluation framework). Evaluations are planned at multiple levels and time frames.

In summary, the protocol is an interventional single-arm multi-centre trial with one year of interventions on multiple levels and four accompanying points of measurement, and a 16-month follow-up. At Current Controlled Trials http://www.controlled-trials.com it was registered as ISRCTN55213782. There is a comprehensive project web site in German http://www.ff100.de, with an English summary http://www.ff100.de/index.php?page=neu.

### Recruitment, inclusion and exclusion at the institutional and at the individual level

**Figure 1:**
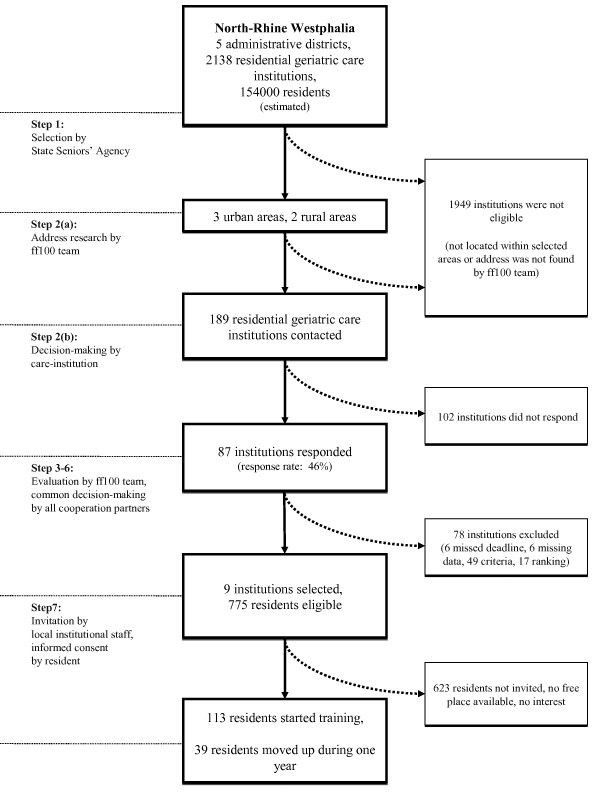
**Flow chart: Recruitment procedure for individuals and institutions**. Each step, including corresponding selection criteria, is described in the subsection Recruitment, inclusion and exclusion at the institutional and at the individual level (Methods). The label *ff100 team *connotes the academic collaborators of the *fit for 100 *project.

The following sections describe the recruitment criteria and procedures. The statements focus at the institutional level (care homes, subsection Procedure of model development) and at the individual level (participants, subsection Recruiting subjects for training groups). Figure 1 (section Preliminary results: establishing model institutions) gives an overview on the selection process, as well as on some resulting numbers of stakeholders at different levels. There were no participants or test persons planned at the political-cultural level. The activities in that context are written down in subsection Intervention).

#### Procedure of model development

The trial is conducted in North-Rhine Westphalia, a German state with around 857,000 inhabitants aged 80 years and over (70% female) [46]. About 485,000 of them are in need of care, nearly 154,000 estimated to live in one of 2,138 residential geriatric care homes [47]. The plan to select candidates for institutional models of this population is as follows:

Step 1. The State Seniors' Agency, an official lobby organisation for the concerns of seniors, preselects five regions, intending to distribute governmental support over the state's five administrative districts.

Step 2. Using a written questionnaire, an ascertainment of all institutions of geriatric care is strived for. The subjects covered are present physical activity offers, interest in the new programme, and general data on the institution and its target group. Before sending the questionnaire, a personal contact is made by phone, in order to prepare participation in the enquiry.

Step 3. According to the aims above, candidates for the model project are detected by an exclusion process with predefined criteria:

- rate of educated nurses lower than 55%,

- less than 60 residents who are not bedridden,

- no staff to guide participants to the training session and back,

- no staff to take part in the project training, and

- not enough room for regular group exercise (e.g. rearranging a dining room is not acceptable).

Step 4. The remaining institutions are ranked along the questionnaire results and the following criteria of general conditions:

- balance between the preselected urban and rural regions,

- enlist ambulant care, day care and residential care,

- enlist different responsible institutions in the health system,

- contain at least some proposals of cooperation partners, and

- include different target groups, e.g. demented seniors.

Step 5. The "top three" in each preselected region are visited by project staff. The following aspects are considered in the visitation and the final selection:

- manifest strong interest in a participation in the project, in further development of existing offers, in the sustainablility beyond the end of the project,

- show an open mind to subjects like prevention, movement or training,

- positive atmosphere of the conversation, enough time and full attention, and

- positive social interaction of the management, staff and residents.

Step 6. After visitation, the ranking in each preselected region is reconsidered and up to ten institutions are selected, covering the five predefined regions. The selection is discussed with the project partners and is adopted concertedly by the management board (see below).

#### Recruiting subjects for training groups

As stated above, a broad inclusion is intended at the individual level. This corresponds to the inclusion procedure, which leaves the invitation of residents to the local staff. Keeping the "step" notation given above, which again serves as a description of figure 1 (section Preliminary results: establishing model institutions), subject recruitment is considered step 7:

Step 7. According to the grant application, two exercise groups in each of the five institutions were planned.

There are rather aspects of recommendation than decision criteria for inclusion:

- Participants (men and women above 80 years of age) should be able to stand with assistance and understand instructions.

- Group integration of a small number of persons not being able to stand up is made possible by alternative exercises.

- Group integration of a small number of persons with orientation disorders is allowed, because an additional assistant is present in the group sessions.

- The size of the groups should range from 8-12 seniors. This results in an expected sample size of 80-120 at the individual level.

The procedure is prepared by oral information in special meetings for residents, relatives and care-givers, and by a specially conceptualised education of training instructors (see subsection Target group orientation and staff (Background)). Residents are invited by nurses and participate on their own decision. New participants would move up if somone leaves the group. All participants have to give informed consent. A resident cannot be invited if

- he/she is not able to take part in a group activity, or

- exercise has been refused for a medical condition (by nurse or clinician).

### Interventions

The interventional activities are grouped to the levels which have been introduced by the conceptual framework (see section Background).

#### Individual level: exercises and social contact

For one year, two training sessions are conducted each week, separated by at least one day. The first group sessions take 30-45 minutes and later they are extended to 60 minutes. Training volume is controlled via the number of different exercises covered (1-10), exercise repetitions per set (1-10) and number of series of all ten exercises (1-2). Training intensity is controlled by individual weight of dumb bells and ankle weights. Details are laid down in the subsection Development of the new exercise programme (Background).

#### Institutional level: Sustainable implementation of training groups

Right from the start of each training group, a positive example should be established for all local stakeholders (e.g. participants, relatives, care staff, management). Therefore, the group sessions are led by project staff or by exercise professionals with a special training in our programme. Subsequent to this demonstrative and motivating start, institutional staff is trained and would take over the group step by step. Supervision and coaching is conducted by project staff. Supervisors ascribe value to certain qualities of the programme in order to keep them, although resources are known to be scarce. Critical qualities, for example, are conduction of two sessions per week instead of one, or the presence of an assistant during group sessions in addition to the instructor.

During this process, the management and director of the institution are continuously provided with information. Other stakeholders (see above) and the physicians in charge are also informed. Conditions and possibilities to resume the training group after the end of the project are discussed. The negotiations would aim at the institution taking over the responsibility for the training group at a certain date, including personnel and finances. The adoption should be completed after 12 months at the latest.

#### Political-cultural level: convincing professionals, administration and the public

Exercise in accordance to training principles, offered to highly aged people in need of care seems strange for parts of the public and also for many professionals and decision-makers in the field of geriatric care and relevant administration. Therefore, activities at the political and cultural level aim at information, persuasion and gaining support for the continuation of the project, namely the training groups of stage I and the conduction of stage II and stage III of the project (see subsection Aims, approaches and strategies (Background)).

Project presentations in professional, administrative and governmental contexts are intended, e.g. during institutionalised meetings and committees, specialised conferences, or fairs. Mostly, attendance to these kinds of meetings is not open. Hence, a special invitation to present the project has to be strived for. Public relation activities (press, TV, Internet) are planned to be carried out as well. While the building of a homepage can be planned and conducted on our own, the attention of press and media has to be attracted, for example on occasion of the start of a new training group.

In addition to these continuing activities, two special milestones have been planned at the end of the stage I project term: first, the publication of a manual for implementing new training groups. Second, the organisation of a public conference with contributions from experts and politics. Last but not least, renowned experts and public figures have been contacted, in order to form an advisory council for all stages of the project, thus reaching out over the edge of stage I, and gaining support for sustainability of the outcomes.

### Types and procedures of data collection

The primary outcome measures directly yield information on the achievement of the aims given in section Aims, approaches and strategies (Background). Measures refering to the effect of the interventions are called secondary outcome measures.

#### Primary outcome measures

At the individual level, the local instructors are told to continuously document individual participation and individual dumb bell and ankle weights. For this purpose, a special form is provided.

At the institutional level, the activities are oriented towards sustainability (see section Aims, approaches and strategies (Background)). Consequently, the success of the efforts will be measured by the number of institutions which will have adopted the training groups after financial support from the project grant has expired. In addition, the duration of funding by the project grant and, where appropriate, the new form of funding will be recorded.

16 months after the time of adoption, follow-up telephone interviews are conducted with the instructors and with the directors of the care residences, in order to learn about maintenance and changes in certain aspects of quality and participation, about problems and possibly about reactions in training and organising the groups. Finances are also covered. Most of the instructors and directors are personally known to the interviewer. There is an emphasis on taking care for a personal atmosphere with an interest in honest and respectful talk, including problems and possible solutions. "Desired" answers should be avoided.

The procedure is as follows: a letter announcing the telephone call would be sent by paper mail. During the second and third week after, the interview would be held. An electronic record of the interview is thought to disturb the open atmosphere. Hence, the interlocutors would be asked to allow a second investigator to listen. If so, the assisting interviewer takes notes of the interview on a prepared scheme with all subjects and questions to be talked of.

At the institutional and political-cultural level, an outcome description is carried out after 12 and 24 months, utilising

• media response (number and types of contributions),

• interest in the website and the offered newsletter (access number and subscription rate),

• efforts to built the advisory council (number and "status" of personalities approached, and of confirmed council members),

• disposition of the grant received and other resources (booking and account data), and

• disposition of personnel costs (analysis of working time).

#### Secondary outcome measures

Effect data are collected at four points in time, at the individual level: just before each group would start and in week 16, 32 and 48. Sensorimotor tests and care-related assessments are selected by reference to everyday life. This should result in high external validity and also increase understanding and motivation of the participants. The institutional documentation of care often seems to contain information on orientation disorders which is not correct or sufficient. Therefore, a screening of cognitive competence by Mini-Mental Status Examination (MMSE) has been included. Details on all tests are given in the following subsections (see also Mechling & Brach [48]).

### Sensorimotor tests

• *Grip strength*. Using a hydraulic hand dynamometer (Jamar, Sammons Preston Rolyan, IL), grip strength indicates general strength [49]. The test person presses the dynamometer with maximum effort. The best out of three attempts with the preferential hand is used.

• *Chair-Stand*. Time is measured for the test person to rise five times from a chair without using the arms. The Chair-Stand-Test [50] is a reliable and feasible test method with high prediction of fall hazards. It is used in many epidemiological studies.

• *Complex rotational flexibility*. This measurement is a combination between trunk and neck flexibility. A specially built device is used [51]. The visual field is wilfully implemented. The tested person has to turn head and trunk as far as possible to look at sports pictograms on a scale behind. Feet are fixed and arms are to be held next to the thighs. The best out of three attempts to either side counts.

• *Shoulder flexibility*. The test person moves the forward-stretched arms upward and backward as far as possible. Shoulder and hip need to touch the back board of the specially built test apparatus [51]. Holding arms vertically above the shoulder joints yields a value of zero. Arms in front of the zero point yield a negative value. Arms behind the zero point yield a positive value.

• *Semi-Tandem-Stand*. Participants should stand with the heel of one foot beneath the big toe of the other foot. This position should be held for 10 seconds without any support. Thus decreased lateral balance control would become obvious. There are clear correlations with falls [52].

• *Soda Pop Test*. Test of hand-eye coordination. The participants have to turn three full soda pop cans placed on a cardboard with their dominant hand. After turning all cans, they have to be reversed the opposite way around. Execution time is recorded in seconds. Procedure and material were slightly modified from Hoeger & Hoeger [[53], pp. 186-187].

• *Colour check test*. This is also an instrument for hand-eye coordination with a higher requirement profile. The test has been adapted from Lemmink and co-workers [54] to frail persons. Four different colours are to be verified and a higher degree of movement accuracy is necessary. 16 coloured tokens (four yellow, four red, four blue and four green) have to be sorted from mixed rows into colour-sorted squares and vice versa. Execution time is measured in seconds. Size and weight of the tokens have been adapted to the grip abilities of frail elderly.

### Care related assessment

• *Barthel Index*. This interview-based disability scale helps to evaluate the self-care ability of patients in ten different areas [55], including feeding, bathing, dressing, walking etc. The abilities are scored from 0 to 15 points per category, depending on the need for assistance. More points mean less needed assistance. Maximum total score is one hundred. 30 or less points mean absolute dependency on care.

• *IADL*. Instrumental Activities of Daily Living (IADL) such as shopping, managing finances, housekeeping and meal preparation etc. describe the functional ability of independent living for elderly persons [56]. Maximum score is eight points. Like Barthel-Index a low score means reduction of independence.

• *Mini-Mental-State-Examination*. The Mini-Mental-State-Examination MMSE was designed as a clinical screening method for grading cognitive impairment [57]. It includes items that assess orientation, registration, recall, attention, calculation, language and visual and constructional tasks. Maximum score is 30 points. A score of 24 or less points can be associated with mental disorders.

• *Individual well-being and expectations*. A questionnaire about current state, subjective feelings of health and well-being, and the expectations upon the intervention programme has been designed. It should also reflect the individual emotional and psychological aspects of the intervention. For all persons with an MMSE score of 24 or less, the individual reference care staff answers the questionnaire.

### Data analyses and evaluation framework

In order to analyse the process and outcomes of the programme, two conceptual frameworks shall be utilised.

For evaluation of the programme, the RE-AIM framework [58,59] seems to be very helpful, especially when the prerequisites and tasks described above (section Aims, approaches and strategies (Background)) are considered. It consists of the dimensions *Reach, Efficacy/effectiveness, Adoption, Implementation *and *Maintenance*, the initial letters forming the acronym RE-AIM. Together they cover the individual and the institutional level. While the *effects *dimension is well-known, an outline on the other aspects follows: *reach *refers to the proportion of the target group reached and whether the participants represent the target group, *adoption *deals with corresponding aspects at the institutional level. *Implementation *describes how often and in what quality the participating institutions deliver the intended service to the target group. Finally, the *maintenance *aspect directly corresponds to one of the aims of the project, dealing with long-time effects at both the individual and institutional level. Data and parameters named in the subsection Types and procedures of data collection (Methods) will be interpreted in the sense of the RE-AIM concept.

Another conceptual framework will be helpful in order to prepare stage III of the project (see section Aims, approaches and strategies (Background)): the programme will be considered an *innovation *in the sense of innovation and diffusion theory [60,61]. Three domains are discriminated in this theory: *invention, testing *and *dissemination*. Assigning costs spent on these domains can help to improve and plan dissemination in stage III.

### Consulting a management board and an ethics committee

A management board has been implemented, including official institutions and project partners: the State Seniors' Agency, and the State Sport Federation of North-Rhine Westphalia. The management board has not only approved before research started, but is consulted on a regular basis (every four months) during the whole project. For example, the preselection of regions, and the final selection of model institutions are discussed in and approved by the management board. The research to be carried out is in compliance with the Helsinki Declaration. The protocol has been fully approved by the German Sports University Cologne Ethics Committee (Cologne, Germany).

### Preliminary results: establishing model institutions

In this section, the results of the selection process are given. The steps are numbered according to the section Recruitment, inclusion and exclusion at the institutional and at the individual level (Methods), where the procedure is described. For an overview, see figure 1.

Step 1. The State Seniors' Agency chose one area in each of the five administrative districts of the state of North-Rhine Westphalia. The result was that the project focussed on three urban and two rural areas.

Step 2. For a complete official directory of geriatric care institutions did not exist, address research on the Internet and in telephone books had to be carried out. A total of 189 institutions were found, between 31 and 48 per area. Reference persons were located and contacted by phone, if possible.

They were presented the rationale of the project and asked for participation. The questionnaire was addressed to the reference person. A person in charge was found for 112 (51%) institutions. The other 77 questionnaires were sent to a general address. For the inquiry happened to take place in the summer vacation season, the response deadline was prolonged and institutions without response were remembered by a phone call.

Step 3. 81 (43%) forms were returned. Six additional returns (3%) missed even the prolonged deadline and could not be considered for reasons of project management. Another six were excluded, because essential data was missing. The remaining 75 forms were evaluated. 49 of them were excluded by the criteria given in the section Recruitment, inclusion and exclusion at the institutional and at the individual level (Methods):

- rate of educated nurses lower than 55% (35),

- less than 60 ambulating residents (10),

- no staff to guide participants to the training session and back (1),

- no staff to take part in the project training (2), and

- not enough room for regular group exercise (1).

Step 4. The remaining 26 institutions were ranked on the given data and the criteria of general conditions (see subsection Procedure of model development (Methods)).

Step 5. Visitations of the 15 candidates, three in each preselected region, were arranged. The visitation included talks of two team members with the director, the managing nurse, the manager of the allied health services, and other key stakeholders. Valuable personal impression was evaluated and a final set of potential models was recommended, according to the given soft criteria (see subsection Procedure of model development (Methods)).

Step 6. A total of nine institutions was recommended for the project. The selection was discussed with the management board and was adopted concertedly. Details can be found in [48].

Step 7. In the model institutions, a total of 775 residents were eligible for participation in the exercise groups. 113 of them started the training. The average of 12.6 participants per group was higher than the recommended group size of 8-12 persons. Despite the number of groups was less than maximum (nine vs. ten), the number of individuals was within the expected range. 39 additional persons moved up, when places were left free. The 152 overall participants were 11.8% male (mean age ± standard deviation: 78.1 ± 11.2 years), 88.2% female (82.2 ± 11.2 years).

## Discussion

We conceptualized a strength and balance programme for very old persons endangered by, or actually in, need of care. Using a multi-centre trial design with one year of interventions at multiple levels, research on feasibility and implementation is started in residential care institutions.

A reason for this is an image seen not only in the public, but - often implicitely - also in health care professionals and decision-makers: residential geriatric care is seen as the endpoint of an intervention continuum covering prevention - acute care - rehabilitation - residential care, the last section mostly being a terminus without return. This image is also reflected in health care systems. Therefore, preventive balance and strength training *within *residential care may sound like an oxymoron to many ears. Consequently, our scientific research in this topic uses a multi-level approach: while literature shows evidence for training effects at the individual also in highest age, feasibility and change management at the institutional level as well as training and behaviour of the instructors should be focus of further research.

Using "an approach of technological science" [11], the trial described here should yield some evidence for implementation and maintenance of the new training programme, and on the long run maybe some ideas for system change management. First results show the feasibilty of the programme, even for residents suffering from severe orientation problems [62,63]. They even seem to benefit to a similar extend [64], a hypothesis subject to planned analyses and further studies.

While our own programme focusses on strength more than on balance, Oddsson and colleagues recently published a programme [65] which targets on balance exercise. Similar to our programme, they attached importance to training principles. Possibly, dependent on the target group, a combination could be encouraged in the future.

## Competing interests

The authors declare that they have no competing interests.

## Authors' contributions

HM and MB were responsible for the identification of the problem, fund-raising, planning and leading the project. UN and FN are executive managers of the project. All four contributed to and take full responsibility of the paper. Applying the co-authorship scoring system published by R. Hunt (Trying an authorship index. Nature 1991, 352(352):187), HM scored highest in the intellectual category (planning, designing, interpreting), MB in the specialist category (input from related fields), and UN and FN in the practical category (data-capture, data processing, organising). Again, all four scored in each category.

## Pre-publication history

The pre-publication history for this paper can be accessed here:

http://www.biomedcentral.com/1471-2318/9/51/prepub
